# Strengthening Early Childhood Protective Factors Through Safe and Supportive Classrooms: Findings from Jump Start + COVID Support

**DOI:** 10.3390/children12070812

**Published:** 2025-06-20

**Authors:** Ruby Natale, Tara Kenworthy LaMarca, Yue Pan, Elizabeth Howe, Yaray Agosto, Rebecca J. Bulotsky-Shearer, Sara M. St. George, Tanha Rahman, Carolina Velasquez, Jason F. Jent

**Affiliations:** 1 Mailman Center for Child Development, School of Medicine, University of Miami Miller, Miami, FL 33136, USA; rnatale@med.miami.edu (R.N.); tlk38@med.miami.edu (T.K.L.); ewfxhowe@gmail.com (E.H.); yagosto@med.miami.edu (Y.A.); txr321@med.miami.edu (T.R.); cxv308@med.miami.edu (C.V.); 2 Department of Public Health Sciences, School of Medicine, University of Miami Miller, Miami, FL 33136, USA; panyue@med.miami.edu (Y.P.); s.stgeorge@med.miami.edu (S.M.S.G.); 3 Department of Psychology, University of Miami, Coral Gables, FL 33146, USA; rshearer@miami.edu

**Keywords:** infant and early childhood mental health consultation, protective factors, behavior problems, social–emotional development, COVID-19

## Abstract

Background/Objectives: Early care and education programs promote children’s social–emotional development, predicting later school success. The COVID-19 pandemic worsened an existing youth mental health crisis and increased teacher stress. Therefore, we applied an infant and early childhood mental health consultation model, Jump Start Plus COVID Support (JS+CS), aiming to decrease behavioral problems in children post-pandemic. Methods: A cluster randomized controlled trial compared JS+CS to an active control, Healthy Caregivers–Healthy Children (HC2), at 30 ECE centers in low-income areas in South Florida. Participants were not blinded to group assignment. Teachers reported on children’s social–emotional development at baseline and post-intervention using the Devereux Early Childhood Assessment and Strengths and Difficulties Questionnaire. We assessed whether teacher stress, classroom practices, and self-efficacy mediated the relationship between JS+CS and child outcomes. We also explored whether baseline behavior problems moderated JS+CS effects on child protective factors, relative to HC2. Results: Direct group-by-time differences between JS+CS and HC2 were limited. However, JS+CS demonstrated significant within-group improvements in teacher-reported child protective factors, behavior support practices, and classroom safety practices. Classroom safety practices consistently mediated positive changes in child behaviors, including the DECA total protective factor score and subdomains of initiative and self-regulation. Additionally, teacher perceptions of behavior support mediated gains in child attachment. Conclusions: JS+CS shows promise in building protective systems around children through intentional support for teachers, underscoring the value of whole-child, whole-environment approaches in early intervention.

## 1. Introduction

Early childhood is a critical period of development, as children’s environments and experiences shape later outcomes [1,2]. Social–emotional development is especially important, as it is a powerful indicator of young children’s future success in school and into adulthood [3,4,5]. Most children spend some time daily in some form of early care and education (ECE) program [6,7], many for at least 35 h per week [8]. These programs foster the learning and development of critical academic and social–emotional skills. However, children with poor or delayed social–emotional skills are more likely to engage in challenging behaviors, which puts them at risk for suspension and expulsion from ECE programs [9,10]. In fact, it is far more likely that children will be suspended from ECE centers than K-12 programs [11].

Attendance in an ECE program is particularly important for young children who are from minoritized racial and ethnic backgrounds, as they may experience higher levels of risk factors related to their development. For example, social environments in which children or families experience racial discrimination can negatively influence both health and behavioral outcomes [12]. Yet, suspensions and expulsions are higher in boys and Black and Hispanic children relative to their White peers [13,14]. The suspension and expulsion of these young children both limit their educational opportunities in the short term and are linked to long-term negative impacts on their social–emotional development and the risk of permanent school dropout years later [15]. It is therefore critical to identify alternative interventions to manage child behaviors in the classroom. Interventions implemented within ECE programs and the systems that surround them, such as high-quality learning programs and infant and early childhood mental health consultation, have demonstrated success in improving outcomes for marginalized youth in multiple domains (e.g., social–emotional, cognitive, and physical) [16,17,18,19].

Efforts to promote young children’s positive social–emotional development have become even more critical post-COVID-19 [20,21]. The initial impact and sequelae of the pandemic, including lockdowns, social isolation, and increased family stressors, have contributed to a crisis in children’s mental health [22] that has persisted in the years post-pandemic [23]. It is therefore imperative to provide ECE programs and teachers with the knowledge and skills for teaching emotional regulation and other resiliency skills during this post-pandemic period [17,24,25].

The COVID-19 pandemic also negatively impacted childcare centers, particularly in poor counties that were epidemic hotspots, like Miami-Dade in Florida [26]. In 2020, a third of Miami’s childcare centers permanently closed [27], which led to an urgent need to help the remaining centers maintain high-quality care while adapting to critical safety requirements [28,29,30]. The Centers for Disease Control and Prevention (CDC) published public health guidelines to improve physical safety, but these guidelines were costly, complex, and frequently changing, leading to implementation barriers for many community-based childcare centers. These additional job demands were among many stressors for early childhood teachers during COVID-19 [31,32], in a profession where burnout and turnover were already common [33]. Furthermore, there are negative outcomes for children when teachers are stressed in ECE settings. For example, workplace stress is related to increased conflict in teacher–child relationships [34], and teacher emotional health and well-being are negatively associated with exclusionary practices [35]. It is therefore essential to support teachers and foster an environment at the center level that promotes growth during stressful times.

Jump Start Plus COVID Support (JS+CS) [36] is an infant and early childhood mental health consultation (IECMHC) program that adapted the Georgetown Model of IECMHC for the COVID-19 pandemic [37,38]. Bronfenbrenner’s Bioecological Systems Theory serves as the foundation for the Georgetown Model of IECMHC. According to this paradigm, children develop within nested environmental systems, such as their families, schools, and larger cultural contexts. The Georgetown Model employs multilevel interventions (center, teacher, and child) that target systemic concerns rather than concentrating only on individual children [39,40]. This strategy, which has been modified for Jump Start, acknowledges that difficult behaviors frequently result from external circumstances, including poor teacher communication, difficulties at the center director level, and/or high teacher turnover brought on by work-related stress. Multilevel interventions that focus on the larger ecological system surrounding the child are therefore required [41]. Within the Jump Start IECMHC model, the teacher’s skills and practices are the focus of the intervention as a means to improve child outcomes. The goal of IECMHC is to increase teacher capacity to manage challenging behaviors and promote social–emotional skills in children [42]. Master-level mental health consultants trained in IECMHC practices meet with teachers on a weekly basis to help them address their professional development goals related to promoting young children’s social competence [38]. There is a strong evidence-base for the effectiveness of IECMHC for improving children’s social–emotional well-being [43] and growing evidence that IECMHC promotes social–emotional learning in both children and their teachers [44]. We expect that children with more behavioral problems will benefit more from the intervention, as high-quality classroom practices offer greater benefits for children with behavioral risk factors [45,46,47]. Yet, the mediating factors associated with child outcomes have not been studied. A 2010 research synthesis called for a better understanding of the extent to which teachers’ skills or classroom practices mediate the relationship between mental health consultation interventions and child outcomes [48]. This information is crucial to understand, especially in the context of public health crises such as COVID-19, so that interventions can be designed to support teachers in keeping children safe.

**Figure 1:**
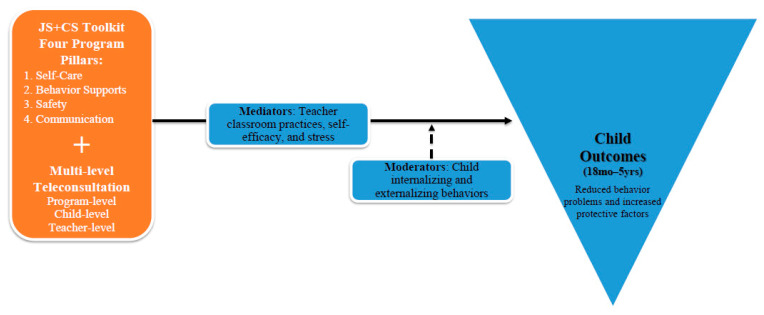
Program model overview.

The purpose of this study was to explore how JS+CS, an IECMHC program adapted for public health crises like COVID-19, improved child social–emotional outcomes. JS+CS was adapted by promoting self-care and improving coping strategies by teachers within childcare centers to support children’s needs during a public health crisis (see Figure 1 for program model overview) [36]. The theoretical underpinnings are based on the Georgetown Model of IECMHC (REF). Based on this framework, we posit that improved child psychosocial functioning (reduced internalizing/externalizing behaviors and increased prosocial behaviors) will improve via improvements in teachers’ practices (e.g., use of trauma-focused behavior management), self-efficacy in handling challenging behaviors (attitudes), and beliefs (support from center directors) [38,49]. Thus we hypothesized that JS+CS would lead to (1) improved child protective factors as measured by the overall social–emotional protective factors on the Devereux Early Childhood Assessment (DECA; comprising three subscales: initiative, attachment, and self-regulation), (2) reduced child problem behaviors, (3) effects of JS+CS on child social–emotional and problem behaviors being mediated by teacher classroom practices and teacher stress, and (4) problem behaviors moderating the relationship between JS+CS practices and child protective factors, relative to an active control group and controlling for sociodemographic characteristics. Our study aimed to answer the following research questions:

1. Was the teacher’s implementation of JS+CS practices effective in increasing children’s protective factors over time, as mediated by teacher stress, teacher self-efficacy, and classroom practices, relative to the active control?
2. Was the teacher’s implementation of JS+CS practices effective in decreasing children’s problematic behaviors over time, as mediated by teacher stress, teacher self-efficacy, and classroom practices, relative to the active control?
3. To what extent do child externalizing and internalizing behaviors at baseline moderate the relationship between JS+CS practices and improvements in children’s protective factors over time, relative to the active control?

## 2. Materials and Methods

### 2.1. Setting

This study took place in 30 ECE centers in South Florida, United States. The center inclusion criteria were as follows: (1) have ≥50 children (≥30 of whom are 18 months–3 years old); (2) be located in the low-income census tract, with at least 50% of families receiving childcare subsidies; (3) serve at least 60% Hispanic or 60% Non-Hispanic Black families; (4) have directors, teachers, and parents who agree to participate; and (5) have no prior enrollment in an early childhood mental health consultation program.

### 2.2. Participants

A total of 608 children and 190 teachers participated in this study (see Table 1 for child demographics and Table 2 for teacher demographics). This study was approved by the university’s Institutional Review Board (IRB) and is currently registered with ClinicalTrials.gov (NCT05445518). The teacher participants and parents/guardians of the child participants signed informed consent forms prior to participating.

### 2.3. Measures

#### 2.3.1. Demographics

Parents completed a 33-item intake form that collected sociodemographic information about themselves and their child. Child-specific items included age, gender, race, ethnicity, English proficiency, preferred language, and health insurance coverage. Teachers completed both an intake form and a classroom demographics form, which collected information about themselves as early childcare professionals, the makeup of their classroom, and suspension and expulsion practices.

#### 2.3.2. Child Protective Factors

The Devereux Early Childhood Assessment (DECA) for Infants and Toddlers (DECA-I/T) [50] and the DECA for Preschoolers, Second Edition (DECA-P2) [51], are validated, reliable parent and teacher report measures of protective factors that promote resilience in children ages 1 month through 5 years [52,53]. In this study, we focus on the teacher report scale. These measures are standardized and norm-referenced. There are 36 items on the DECA-I/T and 38 items on the DECA-P2 that are rated on a 5-point scale from “never” (0) to “very frequently” (4). The measures yield three subscales: initiative (i.e., the ability to use independent thoughts and actions to meet needs), self-regulation (i.e., the ability to express emotions and behaviors in healthy ways), and attachment/relationships (i.e., a mutual, strong, long-lasting relationship between children and significant adults). Example items include “try to clean up after herself/himself”, “adjust to changes in routine”, and “act happy when praised” (DECA-I/T), as well as “show an interest in learning new things”, “show affection for familiar adults”, and “play well with others” (DECA-P2). These measures yield subscale scores as well as a total protective factor (TPF) score. The DECA measures have adequate internal consistency in English- and Spanish-speaking, low-income, and diverse samples [54]. In this study, the internal consistency for the DECA total protective factors scale was a = 0.975.

#### 2.3.3. Child Externalizing and Internalizing Behaviors

The Strengths and Difficulties Questionnaire (SDQ) is a 25-item behavioral screening measure for youth ages 2–17, with versions for 2–4-year-olds and 4–17-year-olds. The SDQ has also shown promising reliability for 12–24-month-old children, though reliability was better for externalizing than internalizing subscales [55]. There are both parent and teacher report versions, and the teacher report is used for this study. Items are rated on a 3-point Likert scale ranging from “not true” (0) to “somewhat true” (1) and “certainly true” (2). The items are divided into five scales in the following domains: emotional symptoms, conduct problems, hyperactivity/inattention, peer relationship problems, and prosocial behavior. Example items include “Often loses temper” (conduct), “Helpful if someone is hurt, upset or feeling ill” (prosocial), and “Many fears, easily scared” (emotional). The conduct problems and hyperactivity/inattention scales are summed for an externalizing score; the emotional and peer problems scales are summed to create an internalizing score. Based on published SDQ scoring categories, children’s scores for total problems, externalizing problems, and internalizing problems were categorized as being within the normal, borderline, high, or very high range. The SDQ has established validity and reliability [56]. In this study, the internal consistency for the SDQ total problem scale was α = 0.860.

#### 2.3.4. Teacher Implementation of JS+CS Model

The Health Environment Rating Scale-Classroom (HERS-C) is a 30 min classroom observation developed by the study investigators that measures the extent to which teachers implement practices and policies within the classroom related to the JS+CS intervention model. The observation comprises four domains: safety, behavioral supports, communication, and resiliency coping. These domains align with core national standards for health and safety in ECE programs [57], map onto the JS+CS pillars, and allow for the measurement of expected areas of change in the control centers’ obesity prevention intervention (nutrition and physical activity). Items reflect key classroom practices such as the “number of times children are taken outside for active play per 8 h day” and how often “high sugar/high fat snacks such as chips, cake, pastry, doughnuts and ice cream are served”. This measure is scored on a 7-point Likert scale rated from “little or no implementation” (1) to “excellent implementation” (7). In this study, internal consistency of the HERS-C subscales ranged from adequate to good, with Cronbach’s a coefficients of 0.62 for safety, 0.85 for behavior management, 0.71 for communication, and 0.70 for resiliency. All four pillars were examined.

#### 2.3.5. Teacher Self-Efficacy

The Teacher Opinion Survey is a 12-item self-report measure of teachers’ confidence in their ability to manage challenging child behaviors [58]. Example items include “If a student in my class became disruptive and noisy, I feel pretty sure that I’d know how to respond effectively” and “I have enough training to deal with almost any classroom situation”. It is rated on a Likert scale from “strongly disagree” (1) to “strongly agree” (5), with higher scores representing higher perceptions of teachers’ confidence in managing children’s behaviors. The total score for this measure in this study had adequate internal consistency (α = 0.785).

The Brief Resilient Coping Scale is a 4-item measure of the tendency to cope with stress in an adaptive manner, with demonstrated reliability and validity [59]. Representative items include “I look for creative ways to alter difficult situations” and “Regardless of what happens to me, I believe I can control my reaction to it”. Items are rated on a 5-point scale from “does not describe me at all” (1) to “describes me very well” (5) and are summed to identify low (4–13 points), medium (14–16 points), and high (17–20 points) resilient copers. In this study, the internal consistency for the Brief Resilient Coping Scale was α = 0.835.

#### 2.3.6. Teacher Stress

The Everyday Stressors Index (ESI) is a 20-item measure of common life stressors such as family, housing, transportation, and employment [60]. Items reflect experiences, for example, “having too many responsibilities” and “not enough money for basic necessities, such as clothing, housing, food, and health care”. The ESI has shown good reliability and validity with low-income and Hispanic populations [61]. It is rated on a Likert scale from “not bothered at all” (1) to “bothered a great deal” (4), with higher ratings indicating more worry, upset, or bother from problems. There was also an option to select “don’t know” (0). In this study, the internal consistency for the Everyday Stressors Index was α = 0.868.

The Child Care Worker Job Stress Inventory is a 51-item measure of workplace stress for childcare center workers, with established reliability and validity [62]. This measure consists of three 17-item scales: job demands, job control, and job resources. The job demand and job resource scales include questions that begin with the stem “How often do the following things happen at work?” which includes “I have to work long hours” and “I feel respected for the work that I do” and are rated on a Likert scale from “rarely/never” (1) to “most of the time” (5). The job control scale items begin with the stem “How much control do you have over the following things at work?” which includes “the types of daily activities that you do” and are rated from “very little” (1) to “very much” (5). In this study, internal consistency of the Childcare Worker Stress Inventory subscales was excellent, with Cronbach’s a coefficients of 0.83 for job demands, 0.87 for job control, and 0.94 for job resources.

### 2.4. Procedures

Details of the study design and procedures have been described elsewhere [17], but briefly, similar to [63], as the intervention is multilevel and center-wide, we randomly assigned centers (N = 24) to one of two groups (intervention or attention control). In cluster randomized controlled trials such as this, clusters (i.e., childcare centers) rather than individuals are randomized to intervention or control groups, and outcomes are measured on individuals within those clusters. The intervention group received the JS+CS program (n = 12). The attention control group (n = 12) was an active, time-matched group that was placed in the HC2 [63] obesity prevention program, a program unrelated to JS+CS. The implementation phase lasted approximately 14 weeks for both groups. Demographic data and outcome measures were collected at baseline and immediately post-intervention via paper surveys or an online database, REDCap [64], based on participant preference. Virtual telepresence robots were utilized to complete classroom observational measures (i.e., HERS-C).

#### 2.4.1. Jump Start Plus COVID Support (JS+CS)

JS+CS is an IECMHC program modeled after Georgetown University’s IECMHC model to improve children’s social, emotional, and behavioral development, prevent behavioral challenges, and reduce suspension and expulsion. JS+CS incorporates *Caring for Our Children National Health and Safety Standards* [57], CDC COVID-19 guidelines for childcare centers [65], and evidence-based practices for building social competence in children [66]. It is organized into four pillars: self-care, trauma-informed behavior support, safety, and communication. It is delivered by mental health consultants at the program (to directors), classroom (to teachers), and child (to parents) levels [36]. Given that the pandemic led childcare centers to restrict visitors at times, consultations were applied in a hybrid model, both in person and via virtual telepresence robotic consultation [63]. In addition, teachers received multiple resources, including 24 infographics covering each of the program’s four pillars.

#### 2.4.2. Healthy Caregivers–Healthy Children (HC2)

HC2 is an existing, evidence-based obesity prevention program that targets childcare centers’ nutrition and physical activity environments and has been delivered by mental health consultants in settings similar to this study [67,68,69]. The HC2 toolkit is organized around four program policies: (1) snack, which emphasizes serving fresh fruits, vegetables, and whole grains over sweets and high-fat foods; (2) beverage, which involves offering low-fat or non-fat milk, limiting juice, and promoting water; (3) physical activity, which encourages providing at least 90 min of physical activity daily; and (4) screen time, which limits screen use to under 30 min per week. Like JS+CS, HC2 was implemented over 14 weeks with a minimum of one hour per week using the same multilevel approach (program, classroom, and child), but was delivered by trained research assistants through in-person visits or virtual telepresence robots. Teachers received weekly lesson plans tailored to each policy, along with Supplementary Materials (e.g., puppets, soccer balls, and parachutes). Control centers received the same pre- and post-intervention measures and incentives as the JS+CS sites to ensure retention.

### 2.5. Analysis

To evaluate the effectiveness of the JS+CS program compared to the HC2 control group, we first conducted generalized estimating equation (GEE) analyses for each child outcome. Seven outcome variables were examined, including four DECA subscales—total protective factors, attachment/relationships, self-regulation, and initiative—and three SDQ subscales: total difficulties and externalizing and internalizing problems. Each outcome was modeled as a function of treatment group (JS+CS vs. HC2), time (baseline vs. follow-up), and their interaction, adjusting for child age. Repeated measures were accounted for using an exchangeable correlation structure, with child ID specified as the repeated subject. These models allowed us to estimate the time-specific effects of JS+CS and determine whether outcomes improved more in the intervention group relative to the control group. We used an intent-to-treat (ITT) approach that retained all participants with baseline data and applied robust standard errors to mitigate potential biases. We also conducted sensitivity analyses by comparing models restricted to complete cases to examine the robustness of our findings.

To investigate potential mechanisms through which JS+CS exerts its effects, we implemented a longitudinal mediation analysis using the GEE framework. For each of the seven outcomes and ten proposed mediators (e.g., teacher stress, teacher efficacy, and classroom practices), we conducted a three-step mediation procedure. Step 1 estimated the total effect (Path C) of JS+CS on the outcome. Step 2 estimated the effect of JS+CS on the mediator (Path A). Step 3 included both the treatment and the mediator to estimate the direct effect of treatment (Path C′) and the mediator’s effect on the outcome (Path B). Indirect effects were computed as the product of coefficients A × B, and standard errors were calculated using the delta method. Mediation was classified as full, partial, or none based on the significance of the indirect effect and comparison between total and direct effects. Full mediation was defined as a statistically significant indirect effect accompanied by a non-significant direct effect (Path C′), indicating that the mediator fully explains the treatment–outcome relationship. Partial mediation was defined as both the indirect and direct effects being significant, suggesting that the mediator accounts for part, but not all, of the intervention’s effect. This longitudinal GEE approach leveraged the repeated measures design to account for within-subject correlations and produce population-averaged estimates.

**Figure 2:**
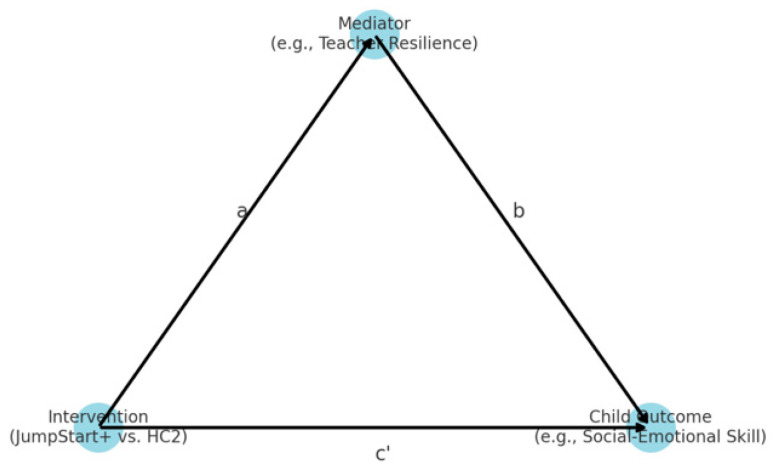
Conceptual mediation model for JS+CS analysis.

In parallel, we conducted a change score-based mediation analysis using linear regression models to examine how changes in mediators contributed to changes in child outcomes (See Figure 2). For each outcome–mediator pair, we computed difference scores from baseline until follow-up. We then estimated Path A (effect of JS+CS on change in the mediator), Path B (effect of mediator change on outcome change, controlling for treatment), and Path C (total effect of JS+CS on outcome change). Indirect effects were calculated as the product of treatment → mediator and mediator → outcome paths.

Significance was determined via Z-tests, and mediation was classified as full, partial, or none depending on whether indirect and/or direct effects were statistically significant. The same definitions of full and partial mediation were applied: full mediation required a significant indirect effect and a non-significant direct effect, while partial mediation required both paths to be significant. This approach provides a straightforward interpretation of change over time, directly focusing on pre–post differences attributable to the intervention. The GEE method leveraged repeated measures for robust population-averaged estimates, while the change score method isolated treatment-driven within-subject differences. Results from both approaches were compared to ensure consistency and to enhance the validity of conclusions regarding mediation.

We also examined potential moderation by baseline SDQ risk categories by including three-way interactions (e.g., treatment × time × SDQ category), with SDQ total, externalizing, and internalizing classifications included as moderators in separate models.

All models reported parameter estimates, robust standard errors, *p*-values, and 95% confidence intervals. All statistical analyses were conducted using SAS (version 9.4), with supplemental data cleaning, visualization, and descriptive statistics conducted in R (version 4.4.2) using the base, Hmisc, dplyr, tidyr, lme4, and emmeans packages [70,71,72,73,74].

## 3. Results

### 3.1. Effectiveness of JS+CS

Analysis of the data revealed several significant findings regarding the treatment groups and changes over time (See Table 3). Although children were randomized to intervention conditions at the childcare center level, teacher reports indicated significant differences in protective factors and problem behaviors between intervention groups. Children in the JS+CS group were rated by teachers as demonstrating significantly lower protective factors on the DECA compared to the HC2 group, indicating worse overall protective factors (β = −5.3576, *p* < 0.0001), attachment/relationships (β = −2.4879, *p* < 0.0001), self-regulation (β = −4.1818, *p* < 0.0001), and initiative (β = −6.5743, *p* < 0.0001). Additionally, the JS+CS group exhibited significantly higher SDQ total problems (estimate = 2.0777, *p* < 0.0001) and externalizing scores (estimate = 1.6939, *p* < 0.0001) relative to the HC2 group, reflecting more problematic behavioral concerns as perceived by their teachers. However, no significant difference was observed between groups on SDQ internalizing scores (β = 0.3805, *p* = 0.0901).

As it relates to within-subject changes over time, both children in the JS+CS and HC2 groups demonstrate significant improvements in DECA attachment/relationships scores (β = 3.8329, *p* < 0.0001), and total protective factors (β = 1.9311, *p* = 0.0309) were observed from baseline to follow-up. Other DECA subscales did not show significant changes over the study period. Further, there were no significant changes over time in teacher perceptions of children’s problem behaviors as measured by the SDQ.

No significant treatment X time interaction effects were detected for any outcome measures. This finding suggests that while overall improvements occurred in some child domains (particularly attachment/relationships), the pattern of change over time was similar for both the JS+CS and HC2 groups, with neither group showing differential rates of improvement.

### 3.2. Mediator Analyses

**Figure 3:**
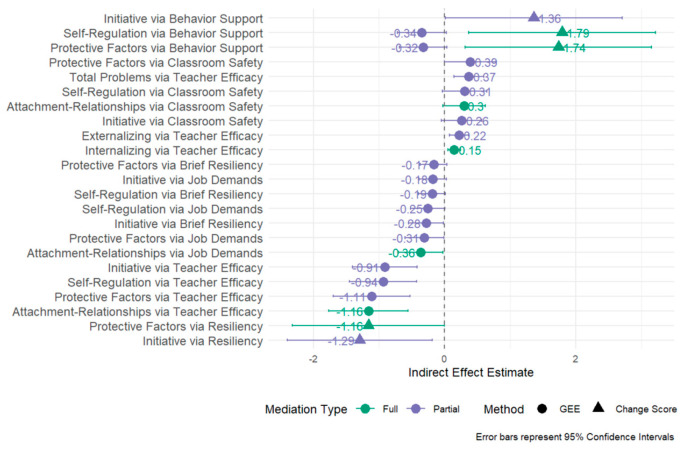
Significant indirect effects of the JS+CS intervention: combined mediation pathways from generalized estimating equations and change score analyses.

Seventy mediation models were tested (see Supplementary Table S1). These models were pre-specified based on the JS+CS conceptual framework, and we hypothesized that multiple teacher-level factors (e.g., stress, self-efficacy, and classroom practices) could independently mediate different child social–emotional outcomes. We used a theory-driven approach to examine all plausible intervention mechanisms, rather than exploratory or data-driven analysis. To enhance clarity and support interpretation, Figure 3 provides a consolidated visual summary of the key mediation pathways identified across all models. These pathways illustrate the extent to which teacher perceptions and observed practices explained the relationship between JS+CS and various child outcomes.

Our analyses revealed that observed teacher safety practices within the classroom significantly mediated the relationship between the JS+CS intervention and multiple child outcomes in the intended direction. Teacher safety practices fully mediated the relationship between JS+CS and teacher perceptions of children’s attachment/relationships (Z = 1.818). Specifically, JS+CS had a significant positive association with teachers’ observed safety practices within classrooms, which in turn increased teachers’ ratings of children’s attachment/relationships. Teacher safety practices also partially mediated the relationships between JS+CS and teacher perceptions of children’s initiative (Z = 1.652) and self-regulation (Z = 1.780). The JS+CS intervention positively influenced safety practices, which subsequently led to improvements in these developmental domains. Similarly, teacher safety practices partially mediated the relationship between JS+CS and teacher perceptions of children’s overall resilience as measured by the DECA total protective factors (Z = 1.973). JS+CS significantly improved observed teacher safety practices within classrooms, which in turn enhanced teachers’ ratings of children’s overall resilience.

Beyond resilience, teacher safety practices partially mediated the relationship between JS+CS and teacher ratings of children’s externalizing behaviors (Z = −1.699). The JS+CS intervention had a positive impact on observed teacher practices related to safety environments (e.g., COVID-19 protocols and self-regulation spaces for children), and these improved safety environments reduced teachers’ ratings of children’s externalizing behavior problems.

Our mediator analyses also revealed several significant mediational pathways between the JS+CS intervention and child outcomes in unintended directions. The mediational effects primarily operated through classroom-level factors, particularly teacher efficacy and job demands.

Teacher efficacy emerged as the most consistent and powerful mediator across multiple outcome domains. The JS+CS intervention was associated with decreased teacher efficacy, which in turn predicted lower scores on all DECA subscales, including attachment/relationships (Z = −3.777), initiative (Z = −3.622), self-regulation (Z = −3.576), and total protective factors (Z = −3.718). Conversely, this reduced teacher efficacy was linked to increased behavioral problems as measured by the SDQ, including externalizing problems (Z = 2.916), internalizing problems (Z = 3.004), and total problems (Z = 3.155).

Childcare worker job demands represented another significant mediational pathway. The JS+CS intervention was associated with increased job demands, which subsequently predicted decreased scores on attachment/relationships (Z = −2.115), initiative (Z = −1.659), self-regulation (Z = −1.902), and total protective factors (Z = −2.005). Additionally, increased job demands were associated with higher levels of externalizing (Z = 1.553) and total behavior problems (Z = 1.605).

Significant partial mediation was observed across several models, including teachers’ reported use of adaptive coping strategies or resilience. For child initiative, the indirect effect through teacher resilience was significant, indicating that lower teacher resilience in the JS+CS group partially explained reduced child initiative (Z = −2.086), self-regulation (Z = −1.805), and total protective factors (Z = −1.607).

Teacher-observed behavior management practices (HERS-C) partially mediated the effects between JS+CS and child self-regulation, total protective factors, and externalizing behaviors. The JS+CS intervention was associated with decreased teacher-observed behavior management practices, which subsequently predicted decreased scores on child self-regulation (Z = −1.820) and total protective factors (Z = −1.758) and higher levels of externalizing behavior problems (Z = 1.572).

No other teacher practices, beliefs, or stress mediated the relationship between JS+CS and child outcomes.

### 3.3. Mediation Analysis: Change Score Models

Among the seventy models tested (see Supplementary Table S2), five demonstrated significant mediation, indicating that changes in specific teacher-related factors explained the impact of the intervention on child emotional and behavioral improvements (see Figure 3). Notably, improvements in teachers’ perceptions of their own behavioral support fully mediated the intervention’s effect on increasing child protective factors (indirect effect = 1.739; SE = 0.725; Z = 2.40; *p* = 0.017), even though the overall treatment effect was not significant. Similarly, teacher ratings of the child’s resiliency mediated the impact on protective factors (indirect effect = −1.161; SE = 0.590; Z = −1.97; *p* = 0.049), also with full mediation. For children’s social relationship strengths, the indirect pathway through increased teacher behavioral support was again significant (indirect effect = 1.793; SE = 0.728; Z = 2.46; *p* = 0.014), reflecting partial mediation. Likewise, for internalizing behavior (emotional distress), changes in teacher-reported behavior and resiliency explained a portion of the intervention’s effect (indirect effects = 1.361 and −1.294, respectively, *p* < 0.05), indicating partial mediation. In contrast, variables such as teacher self-efficacy, teacher stress, and workplace resources or control were not significant mediators in any models.

While most mediators demonstrated consistent effects across both analytic methods, two outcomes, self-regulation and protective factors via behavior support, showed discrepancies between the GEE and change score mediation analyses. Figure 3 serves as a visual synthesis of all 70 mediation models presented in Supplementary Table S1. This figure was designed to highlight the most robust and theoretically meaningful mediation pathways to help readers navigate the complexity of the findings.

In the GEE-based longitudinal models, behavior support was not a statistically significant mediator for either outcome, with indirect effects that were negative and non-significant (self-regulation: indirect effect = −0.345, SE = 0.189, and *p* = 0.069; protective factors: indirect effect = −0.323, SE = 0.184, and *p* = 0.078). In contrast, the change score analyses revealed significant positive mediation for both outcomes (self-regulation: Indirect effect = 1.793, SE = 0.728, and *p* = 0.014; protective factors: indirect effect = 1.739, SE = 0.725, and *p* = 0.017), suggesting that increases in teacher-rated behavior support from baseline until follow-up were associated with improvements in children’s developmental outcomes. These inconsistencies may reflect methodological differences between the approaches, with GEE models capturing population-averaged effects across timepoints, while change score models isolate within-person changes over time. The divergent findings suggest that although population-level effects of behavior support were modest, individual-level improvements in behavior support may play a meaningful role in enhancing child outcomes.

### 3.4. Moderator Analyses

**Figure 4:**
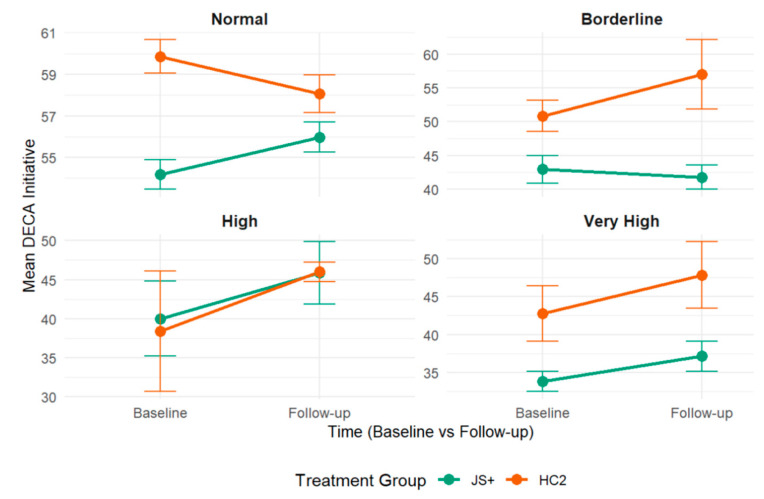
DECA initiative scores by intervention group, stratified by SDQ total problems.

**Figure 5:**
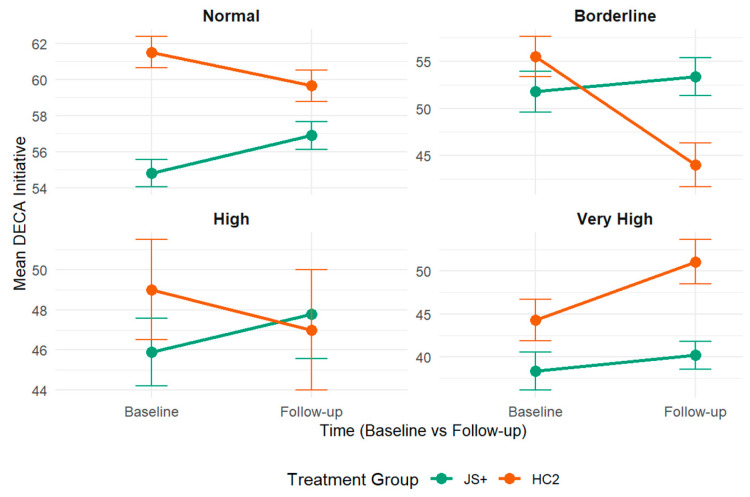
DECA initiative scores by intervention group, stratified by SDQ internalizing problems.

Teacher-reported problematic child behaviors were evaluated as potential moderators of JS+CS intervention effects on children’s protective factors (see Supplementary Table S3 for comprehensive results). Teacher perceptions of children’s total problem behaviors (β = 2.59, SE =1.26, *p* = 0.04; see Figure 4) and children’s internalizing behaviors (β = 3.17, SE =1.27, *p* = 0.01; see Figure 5) significantly moderated the relationship between JS+CS and improvements in children’s DECA initiative outcomes. No other teacher-reported problematic child behaviors significantly moderated the relationship between JS+CS and improvements in other DECA outcomes (i.e., attachment/relationships and self-regulation). Additionally, we conducted moderator analyses to explore whether the effects of JS+CS varied by child gender, race/ethnicity, and language proficiency. No statistically significant three-way interactions were observed in these subgroups.

## 4. Discussion

The purpose of this study was to explore the impact of JS+CS, an IECMHC program adapted for public health crises like COVID-19, on child social–emotional outcomes in ECE settings. Our primary aim was to determine whether JS+CS would improve child protective factors and reduce problem behaviors, as well as to examine whether effects on child outcomes were mediated by teacher stress, self-efficacy, and classroom practices. Additionally, we hypothesized that baseline child behaviors would moderate the relationship between the intervention and outcomes, relative to an active control group. Mediation findings suggested that classroom practices related to safety and behavior support may be critical pathways through which JS+CS influences positive changes in children’s social–emotional development. Findings, though modest, indicate that the JS+CS approach—emphasizing reflective consultation and classroom-based support—may contribute to meaningful changes in early learning environments, even amid implementation challenges and the ongoing impact of the COVID-19 pandemic. Although there were limited between-group effects, the results of this study offer several insights into the effectiveness of JS+CS, as well as areas for further refinement.

### 4.1. Child Protective Factors Mediated by Teacher Stress, Classroom Practices, and Self-Efficacy

Teacher implementation of JS+CS practices had a positive effect on children’s protective factors over time, though the magnitude of the effect was modest. Mediation analysis revealed that improvements in teacher practices, particularly those related to classroom safety practices and behavior support, were key mechanisms through which the intervention influenced child protective factors.

However, while these findings suggest that JS+CS has the potential to enhance social–emotional development in children, the main effects on child outcomes of assignment to the JS+CS intervention condition compared to the HC2 active control condition were not observed. The limited between-group effects may be partially explained by the absence of statistically significant group-by-time interaction effects, which suggests that improvements in child outcomes occurred similarly across both JS+CS and HC2 conditions. This may reflect a limitation in statistical power due to attrition and differential dropout at follow-up. Alternatively, it is possible that both interventions, despite their differing emphases, had similar impacts on child social–emotional development. Given that HC2, while focused on physical health, also provided consistent support during a high-stress period, it may have exerted beneficial effects that narrowed observable differences between groups. It is possible that the initial differences in baseline characteristics between the two groups, despite randomization at the center level, may have masked the impact of the intervention. In addition, teachers in the JS+CS centers may have had a heightened awareness of children’s behavioral difficulties due to the program’s emphasis on mental health consultation, thereby influencing their ratings. The reflective coaching approach employed in JS+CS may have resulted in lower reported teacher self-efficacy, as consultants focused on fostering insight and autonomy rather than providing directive strategies. Research shows that teachers may overestimate their abilities relative to perceptions of their supervisors [75], and therefore, follow-up assessments after reflective supervision may offer a more accurate reflection of teachers’ classroom confidence. Additionally, teachers in the JS+CS condition reported higher job demands, likely due to the intervention’s more intensive structure and the time commitment required for consultation. In contrast, the HC2 intervention included more structured lesson plans and was easier to implement, which may have contributed to its relatively higher feasibility and lower burden on teachers. Given the limited between-group effects, further refinements to the JS+CS intervention, such as a more structured, versus reflective, approach, may be needed to see more pronounced effects on child outcomes.

### 4.2. Children’s Problematic Behaviors Mediated by Teacher Stress, Classroom Practices, and Self-Efficacy

The study found that JS+CS led to reductions in children’s externalizing behaviors over time, which were mediated through changes in classroom safety practices. These mediation results suggest that the JS+CS intervention has the potential to decrease problematic behaviors by improving classroom safety strategies. However, the effect sizes were modest, indicating that while there was a positive trend, the intervention may need to be adapted to provide more continuous access to resources for teachers to achieve more significant reductions in child behavior problems. For example, a mobile application that offers access to ongoing resources with quick accessibility may be more effective in reducing job demands [76].

Another consideration is the differential focus of the two interventions. Even though the JS+CS intervention and HC2 are designed to have differential outcomes, other studies have found that pediatric nutrition and physical wellness interventions for young children can also have a benefit on children’s psychosocial outcomes [77]. While JS+CS centered on social–emotional learning and mental health consultation, HC2 targeted physical health behaviors, such as nutrition and physical activity, which have also been linked to cognitive and behavioral benefits [69].

Timing may have also played a role. The JS+CS and HC2 interventions were delivered during the COVID-19 pandemic, a period in which any form of professional support may have been perceived as particularly valuable. The HC2 model may have been equally effective as JS+CS in supporting teacher behaviors during the pandemic, and this indirectly benefited children’s socio-emotional outcomes, regardless of its primary nutrition and wellness focus.

### 4.3. Children’s Protective Factors Moderated by Children’s Externalizing and Internalizing Behaviors

Some child baseline problem behaviors moderated the relationship between JS+CS implementation and improvements in child social–emotional protective factors. For children exhibiting higher levels of internalizing behaviors at baseline, assignment to the JS+CS intervention condition increased scores on initiative compared to children with similar levels of problem behaviors in the HC2 control group. For children who exhibited lower levels of total problem scores, JS+CS increased children’s initiative compared to children with similar levels of total problem scores in the HC2 control condition. Teachers in JS+CS, compared to teachers in HC2, were able to more effectively utilize the intervention strategies to improve social–emotional outcomes in children who started with higher levels of internalizing problem behaviors. This suggests that JS+CS may be particularly beneficial for children who are at greater risk for developmental delays in social–emotional skills. However, even in these cases, the effect sizes remained modest, suggesting that while the intervention holds promise for children with more pronounced behavioral challenges, additional support or intensified interventions may be necessary for larger and more consistent outcomes. It is also important to note that baseline levels of behavior problems were relatively low across the sample, possibly limiting the potential for observable improvements. In addition, the DECA measure may show natural improvement over time as children mature and adapt to classroom environments, and it therefore may not be reflective of the intervention outcomes.

### 4.4. Limitations

Several limitations should be considered when interpreting these findings. While childcare sites were randomized, teachers in the JS+CS condition rated children as exhibiting more behavioral challenges and fewer protective factors at baseline, which may have influenced the results despite rigorous analytic controls. Future studies should examine additional baseline characteristics to determine potential confounding factors. Additionally, missing data was relatively high—a common issue in community-based effectiveness trials—but intent-to-treat analyses were used to retain as much data as possible. It is also important to acknowledge that teacher-reported measures may pose potential biases, as reflected in the literature [78]. We would also use caution in generalizing the findings outside of Miami-Dade County.

Another important consideration is the potency of the active control condition. HC2 may have been more effective than anticipated, especially during the COVID-19 pandemic, providing meaningful support that narrowed the gap between groups. Consultant turnover was also more pronounced in JS+CS, potentially impacting the consistency and quality of intervention delivery. Finally, long-term follow-ups are needed to assess whether JS+CS may yield delayed benefits that were not captured within this study’s timeframe.

### 4.5. Future Directions

Despite these challenges, JS+CS still demonstrated value in specific domains, and the findings offer direction for future iterations of research. As the field moves into the post-pandemic era, future work should explore how to deliver JS+CS in a way that reduces teacher burden and maintains implementation fidelity, potentially through more streamlined or app-based models. Examining center-level outcomes may also help elucidate how contextual factors influence teacher–child dynamics. Additionally, future studies should explore subgroup effects in greater depth to determine whether specific populations, such as children of different racial/ethnic backgrounds, gender identities, or baseline symptom levels, benefit differentially from the JS+CS intervention. It will also be critical to investigate whether JS+CS yields stronger outcomes in non-pandemic conditions, as earlier studies [79] suggested more promising results. Finally, future studies should examine how fidelity moderates program impact and assess whether observed trends persist or evolve over longer-term follow-ups. Given multiple significant relationships between teacher factors and child outcomes, these outcomes can inform state and federal policies by reinforcing the need to fund and scale ECMHC services within childcare center licensing regulations. Policymakers could leverage JS+CS as a model to support legislation that requires or incentivizes access to mental health consultation in childcare centers, particularly in under-resourced and high-risk communities, as a means to promote equity and reduce disproportionality in early childhood disciplines.

## 5. Conclusions

These findings highlight the complexity of implementing mental health interventions in ECE settings, particularly in the aftermath of a public health crisis. Despite the modest effect sizes, the mediation results provide a promising indication that teacher practices, particularly safety and behavior supports, are key pathways through which the JS+CS intervention positively impacted child social–emotional development. Both JS+CS and HC2 resulted in some improvements in certain social–emotional outcomes, underscoring the potential of these interventions in enhancing children’s well-being. Future research should explore hybrid approaches that integrate both social–emotional learning and physical health promotion to create more comprehensive support systems for young children. Providing more robust professional development, ongoing coaching, and continuous access to resources may help educators manage their own well-being while fostering positive child outcomes. The promising mediation results suggest that with continued refinement and support, interventions like JS+CS have the potential to create lasting improvements in both teacher practices and child social–emotional development.

## Figures and Tables

**Table 1:** Child demographic characteristics by intervention group.

Characteristic	JS+CS (*n* = 287)	HC2 (*n* = 317)	Total (N = 608)	Test Statistic	*p*-Value
Age (years)					
Mean (SD)	3.59 (1.18)	3.34 (1.15)	3.46 (1.17)	*F*(1, 606) = 7.37	0.007
Gender				χ^2^(1) = 7.23	0.007
Female	160 (55.7%)	142 (44.8%)	302 (50.0%)		
Male	127 (44.3%)	175 (55.2%)	302 (50.0%)		
Race				χ^2^(5) = 13.25	0.021
White	206 (74.1%)	198 (64.5%)	404 (69.1%)		
Black	48 (17.3%)	87 (28.3%)	135 (23.1%)		
Native	4 (1.4%)	4 (1.3%)	8 (1.4%)		
American					
Asian Pacific	1 (0.4%)	0 (0.0%)	1 (0.2%)		
Islander					
Multiracial	14 (5.0%)	9 (2.9%)	23 (3.9%)		
Other	5 (1.8%)	9 (2.9%)	14 (2.4%)		
Ethnicity				χ^2^(4) = 20.62	<0.001
Hispanic	238 (83.8%)	217 (68.9%)	455 (76.0%)		
Non-Hispanic	11 (3.9%)	16 (5.1%)	27 (4.5%)		
White					
Non-Hispanic	24 (8.5%)	60 (19.0%)	84 (14.0%)		
Black					
Haitian	7 (2.5%)	18 (5.7%)	25 (4.2%)		
Other	4 (1.4%)	4 (1.3%)	8 (1.3%)		
English Proficiency				χ^2^(1) = 4.90	0.027
Yes	174 (61.5%)	218 (70.1%)	392 (66.0%)		
No	109 (38.5%)	93 (29.9%)	202 (34.0%)		
Primary Language				χ^2^(2) = 16.63	<0.001
English	90 (31.6%)	151 (47.6%)	241 (40.0%)		
Spanish	194 (68.1%)	164 (51.7%)	358 (59.5%)		
Creole	1 (0.4%)	2 (0.6%)	3 (0.5%)		
Secondary Language				χ^2^(3) = 8.45	0.038
English	173 (65.5%)	149 (55.6%)	322 (60.5%)		
Spanish	80 (30.3%)	95 (35.4%)	175 (32.9%)		
Creole	8 (3.0%)	14 (5.2%)	22 (4.1%)		
Other	3 (1.1%)	10 (3.7%)	13 (2.4%)		

Note: Percentages for each characteristic are calculated within treatment groups. JS+CS = intervention group; HC2 = Healthy Caregivers–Healthy Children comparison group. Some categories may not sum to the total N due to missing data.

**Table 2:** Teacher demographic characteristics by treatment group.

Characteristic	JS+CS (n = 86)	HC2 (n = 104)	Total (n = 190)	Test Statistic	*p*-Value
Age (years)					
Mean (SD)	46.09 (12.58)	42.94 (13.67)	44.39 (13.24)	*F*(1, 184) = 2.65	0.105
Gender				χ^2^(1) = 0.06	0.811
Female	84 (97.7%)	101 (97.1%)	185 (97.4%)		
Male	2 (2.3%)	3 (2.9%)	5 (2.6%)		
Race				χ^2^(4) = 1.71	0.79
White	66 (76.7%)	74 (72.5%)	140 (74.5%)		
Black	12 (14.0%)	21 (20.6%)	33 (17.6%)		
Native American	1 (1.2%)	1 (1.0%)	2 (1.1%)		
Multiracial	4 (4.7%)	3 (2.9%)	7 (3.7%)		
Other	3 (3.5%)	3 (2.9%)	6 (3.2%)		
Ethnicity				χ^2^(2) = 10.03	0.007
Hispanic	76 (90.5%)	81 (77.9%)	157 (83.5%)		
Non-Hispanic	1 (1.2%)	7 (6.7%)	8 (4.3%)		
White					
Non-Hispanic	3 (3.6%)	9 (8.7%)	12 (6.4%)		
Black					
Haitian	0 (0.0%)	5 (4.8%)	5 (2.7%)		
Other	4 (4.8%)	2 (1.9%)	6 (3.2%)		
English Proficiency				χ^2^(1) = 6.53	0.011
Yes	32 (37.2%)	57 (55.9%)	89 (47.3%)		
No	54 (62.8%)	45 (44.1%)	99 (52.7%)		
Secondary Language				χ^2^(3) = 6.74	0.081
English	48 (64.0%)	44 (51.2%)	92 (57.1%)		
Spanish	21 (28.0%)	28 (32.6%)	49 (30.4%)		
Creole	0 (0.0%)	6 (7.0%)	6 (3.7%)		
Other	6 (8.0%)	8 (9.3%)	14 (8.7%)		
Education Level				χ^2^(7) = 6.86	0.443
Elementary or	0 (0.0%)	1 (1.0%)	1 (0.5%)		
Less					
Some High School	2 (2.4%)	3 (2.9%)	5 (2.7%)		
High School/GED	16 (19.5%)	19 (18.4%)	35 (18.9%)		
Technical	9 (11.0%)	4 (3.9%)	13 (7.0%)		
Training					
Some College	15 (18.3%)	21 (20.4%)	36 (19.5%)		
Associate Degree	8 (9.8%)	14 (13.6%)	22 (11.9%)		
Bachelor’s Degree	25 (30.5%)	37 (35.9%)	62 (33.5%)		
Graduate Degree	7 (8.5%)	4 (3.9%)	11 (5.9%)		
Professional Experience	*M* (*SD*)	*M* (*SD*)	*M* (*SD*)		
Years as Childcare Professional	11.30 (10.18)	11.24 (8.40)	11.27 (9.17)	*F*(1, 162) = 0.00	0.967
Years at Current Program	8.59 (8.75)	6.28 (7.01)	7.28 (7.87)	*F*(1, 139) = 3.03	0.084
Children Enrolled in Classroom	13.39 (6.15)	12.13 (5.61)	12.70 (5.88)	*F*(1, 187) = 2.14	0.145

Note: Percentages are calculated within intervention groups. JS+CS = intervention group; HC2 = comparison group. Sample sizes vary by characteristic due to missing data.

**Table 3:** Child outcome measures by intervention condition at baseline and follow-up.

	JS+CS (Intervention)		HC2 (Comparison)		Effect	Estimate	Std. Error	*p*-Value
	Baseline (N = 304)	Follow-Up (N = 304)	Baseline (N = 367)	Follow-Up (N = 367)				
DECA Total Protective Factors								
Mean (SD)	51.5 (10.7)	53.1 (10.1)	55.6 (12.5)	57.1 (10.5)	Treatment (JS+CS)	−5.3576	1.065	<0.0001
Missing	82 (27.0%)	104 (34.2%)	137 (37.3%)	213 (58.0%)	Time (Follow-Up vs. Baseline)	1.9311	0.8948	0.0309
					Interaction (JS+CS × Follow-Up)	0.199	1.2054	0.8689
DECA Attachment/Relationships								
Mean (SD)	49.3 (11.2)	51.8 (9.58)	50.5 (11.9)	53.7 (10.5)	Treatment (JS+CS)	−2.4879	0.9724	0.0105
Missing	67 (22.0%)	90 (29.6%)	116 (31.6%)	187 (51.0%)	Time (Follow-Up vs. Baseline)	3.8329	0.818	<0.0001
					Interaction (JS+CS × Follow-Up)	−0.864	1.1522	0.4533
DECA Self-Regulation								
Mean (SD)	52.8 (10.6)	52.9 (10.1)	56.2 (11.9)	56.0 (10.6)	Treatment (JS+CS)	−4.1818	1.0208	<0.0001
Missing	64 (21.1%)	88 (28.9%)	126 (34.3%)	192 (52.3%)	Time (Follow-Up vs. Baseline)	0.3181	0.8269	0.7004
					Interaction (JS+CS × Follow-Up)	0.1974	1.1162	0.8596
DECA Initiative								
Mean (SD)	51.2 (10.9)	53.4 (10.7)	56.7 (12.0)	56.7 (10.5)	Treatment (JS+CS)	−6.5743	1.0586	<0.0001
Missing	70 (23.0%)	91 (29.9%)	119 (32.4%)	196 (53.4%)	Time (Follow-Up vs. Baseline)	0.606	0.8869	0.4945
					Interaction (JS+CS × Follow-Up)	1.9162	1.1374	0.0921
SDQ Total Problems								
Mean (SD)	7.99 (5.88)	7.79 (5.95)	6.65 (6.18)	5.85 (5.87)	Treatment (JS+CS)	2.0777	0.5232	<0.0001
Missing	74 (24.3%)	88 (28.9%)	101 (27.5%)	181 (49.3%)	Time (Follow-Up vs. Baseline)	−0.4308	0.4098	0.2932
					Interaction (JS+CS × Follow-Up)	−0.0408	0.5574	0.9416
SDQ Externalizing								
Mean (SD)	5.36 (4.25)	5.18 (4.08)	4.13 (4.07)	3.69 (3.86)	Treatment (JS+CS)	1.6939	0.3659	<0.0001
Missing	74 (24.3%)	88 (28.9%)	101 (27.5%)	181 (49.3%)	Time (Follow-Up vs. Baseline)	−0.1869	0.289	0.5178
					Interaction (JS+CS × Follow-Up)	−0.2006	0.3936	0.6102
SDQ Internalizing								
Mean (SD)	2.63 (2.57)	2.62 (2.51)	2.53 (2.69)	2.16 (2.60)	Treatment (JS+CS)	0.3805	0.2245	0.0901
Missing	74 (24.3%)	88 (28.9%)	101 (27.5%)	181 (49.3%)	Time (Follow-Up vs. Baseline)	−0.2309	0.1903	0.225
					Interaction (JS+CS × Follow-Up)	0.1561	0.2568	0.5433

Note: DECA = Devereux Early Childhood Assessment; SDQ = Strengths and Difficulties Questionnaire. Higher scores on DECA scales indicate more positive developmental outcomes; higher scores on SDQ scales indicate more problematic behaviors. Statistical effects are derived from mixed-effects models.

## Data Availability

Requests for data can be sent to the corresponding author.

## Supplementary Materials

The following supporting information can be downloaded at https://www.mdpi.com/article/10.3390/children12070812/s1: Table S1: Complete mediation analysis of JS+CS intervention effects on child outcomes via classroom- and teacher-level factors (GEE methods); Table S2: Mediation analysis of JS+CS intervention effects on child outcomes via classroom- and teacher-level factors (change score methods); Table S3: Generalized estimating equation (GEE) models examining the moderating role of SDQ risk categories on intervention effects for DECA outcomes.

## Abbreviations

The following abbreviations are used in this manuscript:

CDC	Centers for Disease Control and Prevention
DECA	Devereux Early Childhood Assessment
ECE	Early care and education
GEE	Generalized estimating equation
HC2	Healthy Caregivers–Healthy Children
HERS-C	Health Environment Rating Scale-Classroom
IECMHC	Infant and early childhood mental health consultation
IRB	Institutional Review Board
JS+CS	Jump Start Plus COVID Support
SDQ	Strengths and Difficulties Questionnaire
