# Supplementary material for: Strengthening Early Childhood Protective Factors Through Safe and Supportive Classrooms: Findings from Jump Start + COVID Support
Source: Children (Basel). 2025 Jun 20;12(7):812. doi: 10.3390/children12070812 (PMC12293144; doi:10.3390/children12070812)
Supplement: Supplementary file 1 [file children-12-00812-s001.zip › children-3646120-supplementary.pdf]

Supplementary Table S1. Complete mediation analysis of JS+CS intervention effects on child outcomes via classroom- and teacher-level factors (GEE methods).

Outcome	Mediator	Path A Est. (SE)	Path B Est. (SE)	Direct Effect (Path C) Est. (SE)	Indirect Effect Est. (SE)	Z-Indirect	Mediation Type
**DECA Attachment/ Relationships**							
	Brief Resiliency	-0.460 (0.176)**	0.024 (0.171)	-1.132 (1.029)	-0.011 (0.079)	-0.140	No Mediation
	CWSI Job Control	-0.088 (0.063)	1.439 (0.520)**	-1.132 (1.029)	-0.127 (0.102)	-1.242	No Mediation
	CWSI Job Demands	0.156 (0.064)*	-2.332 (0.549)***	-1.132 (1.029)	-0.364 (0.172)*	-2.115	Full Mediation
	CWSI Job Resources	0.040 (0.043)	2.079 (0.782)**	-1.132 (1.029)	0.084 (0.094)	0.887	No Mediation
	ESI Teacher	0.352 (1.069)	-0.023 (0.037)	-1.132 (1.029)	-0.008 (0.028)	-0.292	No Mediation
	HERS-C Behavior	-0.168 (0.065)**	1.374 (0.761)†	-1.132 (1.029)	-0.231 (0.156)	-1.484	No Mediation
	HERS-C Communication	0.042 (0.072)	-0.044 (0.619)	-1.132 (1.029)	-0.002 (0.026)	-0.071	No Mediation
	HERS-C Resiliency	-0.645 (0.091)***	-0.312 (0.406)	-1.132 (1.029)	0.201 (0.264)	0.764	No Mediation
	HERS-C Safety	0.135 (0.054)*	2.230 (0.834)**	-1.132 (1.029)	0.300 (0.165)†	1.818	Full Mediation
	Teacher Efficacy	-2.433 (0.557)***	0.477 (0.063)***	-1.132 (1.029)	-1.161 (0.307)***	-3.777	Full Mediation
**DECA Initiative**							
	Brief Resiliency	-0.460 (0.176)**	0.614 (0.178)***	-5.552 (1.028)***	-0.282 (0.135)*	-2.086	Partial Mediation
	CWSI Job Control	-0.088 (0.063)	1.930 (0.507)***	-5.552 (1.028)***	-0.170 (0.130)	-1.306	No Mediation
	CWSI Job Demands	0.156 (0.064)*	-1.134 (0.501)*	-5.552 (1.028)***	-0.177 (0.107)†	-1.659	Partial Mediation
	CWSI Job Resources	0.040 (0.043)	1.649 (0.794)*	-5.552 (1.028)***	0.066 (0.077)	0.857	No Mediation
	ESI Teacher	0.352 (1.069)	0.017 (0.033)	-5.552 (1.028)***	0.006 (0.021)	0.276	No Mediation
	HERS-C Behavior	-0.168 (0.065)**	0.686 (0.788)	-5.552 (1.028)***	-0.116 (0.140)	-0.826	No Mediation

Outcome	Mediator	Path A Est. (SE)	Path B Est. (SE)	Direct Effect (Path C) Est. (SE)	Indirect Effect Est. (SE)	Z-Indirect	Mediation Type
	HERS-C Communication	0.042 (0.072)	-0.233 (0.652)	-5.552 (1.028)***	-0.010 (0.032)	-0.304	No Mediation
	HERS-C Resiliency	-0.645 (0.091)***	-0.108 (0.404)	-5.552 (1.028)***	0.069 (0.261)	0.267	No Mediation
	HERS-C Safety	0.135 (0.054)*	1.923 (0.868)*	-5.552 (1.028)***	0.259 (0.157)†	1.652	Partial Mediation
	Teacher Efficacy	-2.433 (0.557)***	0.375 (0.058)***	-5.552 (1.028)***	-0.911 (0.252)***	-3.622	Partial Mediation
**DECA Self-Regulation**							
	Brief Resiliency	-0.460 (0.176)**	0.413 (0.165)*	-3.121 (1.011)**	-0.190 (0.105)†	-1.805	Partial Mediation
	CWSI Job Control	-0.088 (0.063)	1.394 (0.516)**	-3.121 (1.011)**	-0.123 (0.099)	-1.236	No Mediation
	CWSI Job Demands	0.156 (0.064)*	-1.625 (0.535)**	-3.121 (1.011)**	-0.254 (0.133)†	-1.902	Partial Mediation
	CWSI Job Resources	0.040 (0.043)	3.007 (0.713)***	-3.121 (1.011)**	0.121 (0.132)	0.918	No Mediation
	ESI Teacher	0.352 (1.069)	-0.037 (0.038)	-3.121 (1.011)**	-0.013 (0.042)	-0.312	No Mediation
	HERS-C Behavior	-0.168 (0.065)**	2.045 (0.803)*	-3.121 (1.011)**	-0.345 (0.189)†	-1.820	Partial Mediation
	HERS-C Communication	0.042 (0.072)	0.534 (0.586)	-3.121 (1.011)**	0.022 (0.046)	0.490	No Mediation
	HERS-C Resiliency	-0.645 (0.091)***	0.433 (0.407)	-3.121 (1.011)**	-0.279 (0.265)	-1.053	No Mediation
	HERS-C Safety	0.135 (0.054)*	2.286 (0.895)*	-3.121 (1.011)**	0.308 (0.173)†	1.780	Partial Mediation
	Teacher Efficacy	-2.433 (0.557)***	0.385 (0.062)***	-3.121 (1.011)**	-0.937 (0.262)***	-3.576	Partial Mediation
**DECA Total Protective Factors**							
	Brief Resiliency	-0.460 (0.176)**	0.363 (0.178)*	-4.090 (1.071)***	-0.167 (0.104)†	-1.607	Partial Mediation
	CWSI Job Control	-0.088 (0.063)	1.796 (0.530)***	-4.090 (1.071)***	-0.158 (0.123)	-1.286	No Mediation
	CWSI Job Demands	0.156 (0.064)*	-1.958 (0.556)***	-4.090 (1.071)***	-0.306 (0.153)*	-2.005	Partial Mediation

Outcome	Mediator	Path A Est. (SE)	Path B Est. (SE)	Direct Effect (Path C) Est. (SE)	Indirect Effect Est. (SE)	Z-Indirect	Mediation Type
	CWSI Job Resources	0.040 (0.043)	2.511 (0.809)**	-4.090 (1.071)***	0.101 (0.112)	0.900	No Mediation
	ESI Teacher	0.352 (1.069)	-0.018 (0.038)	-4.090 (1.071)***	-0.006 (0.024)	-0.270	No Mediation
	HERS-C Behavior	-0.168 (0.065)**	1.915 (0.804)*	-4.090 (1.071)***	-0.323 (0.184)†	-1.758	Partial Mediation
	HERS-C Communication	0.042 (0.072)	0.250 (0.619)	-4.090 (1.071)***	0.011 (0.032)	0.332	No Mediation
	HERS-C Resiliency	-0.645 (0.091)***	0.200 (0.421)	-4.090 (1.071)***	-0.129 (0.272)	-0.474	No Mediation
	HERS-C Safety	0.135 (0.054)*	2.909 (0.893)***	-4.090 (1.071)***	0.392 (0.199)*	1.973	Partial Mediation
	Teacher Efficacy	-2.433 (0.557)***	0.458 (0.065)***	-4.090 (1.071)***	-1.113 (0.299)***	-3.718	Partial Mediation
**SDQ Externalizing Problems**							
	Brief Resiliency	-0.460 (0.176)**	-0.118 (0.066)†	1.334 (0.368)***	0.054 (0.037)	1.473	No Mediation
	CWSI Job Control	-0.088 (0.063)	0.272 (0.181)	1.334 (0.368)***	-0.024 (0.023)	-1.020	No Mediation
	CWSI Job Demands	0.156 (0.064)*	0.415 (0.206)*	1.334 (0.368)***	0.065 (0.042)†	1.553	Partial Mediation
	CWSI Job Resources	0.040 (0.043)	-0.609 (0.367)†	1.334 (0.368)***	-0.024 (0.030)	-0.818	No Mediation
	ESI Teacher	0.352 (1.069)	0.029 (0.012)*	1.334 (0.368)***	0.010 (0.031)	0.326	No Mediation
	HERS-C Behavior	-0.168 (0.065)**	-0.560 (0.284)*	1.334 (0.368)***	0.094 (0.060)†	1.572	Partial Mediation
	HERS-C Communication	0.042 (0.072)	-0.036 (0.269)	1.334 (0.368)***	-0.002 (0.012)	-0.132	No Mediation
	HERS-C Resiliency	-0.645 (0.091)***	0.009 (0.157)	1.334 (0.368)***	-0.006 (0.101)	-0.055	No Mediation
	HERS-C Safety	0.135 (0.054)*	-0.736 (0.316)*	1.334 (0.368)***	-0.099 (0.058)†	-1.699	Partial Mediation
	Teacher Efficacy	-2.433 (0.557)***	-0.091 (0.023)***	1.334 (0.368)***	0.222 (0.076)**	2.916	Partial Mediation
**SDQ Internalizing Problems**							

Outcome	Mediator	Path A Est. (SE)	Path B Est. (SE)	Direct Effect (Path C) Est. (SE)	Indirect Effect Est. (SE)	Z-Indirect	Mediation Type
	Brief Resiliency	-0.460 (0.176)**	-0.042 (0.039)	0.153 (0.233)	0.019 (0.019)	0.998	No Mediation
	CWSI Job Control	-0.088 (0.063)	0.001 (0.114)	0.153 (0.233)	0.000 (0.010)	-0.010	No Mediation
	CWSI Job Demands	0.156 (0.064)*	0.239 (0.124)†	0.153 (0.233)	0.037 (0.025)	1.517	No Mediation
	CWSI Job Resources	0.040 (0.043)	-0.264 (0.187)	0.153 (0.233)	-0.011 (0.014)	-0.783	No Mediation
	ESI Teacher	0.352 (1.069)	0.005 (0.009)	0.153 (0.233)	0.002 (0.006)	0.288	No Mediation
	HERS-C Behavior	-0.168 (0.065)**	-0.207 (0.166)	0.153 (0.233)	0.035 (0.031)	1.122	No Mediation
	HERS-C Communication	0.042 (0.072)	0.181 (0.150)	0.153 (0.233)	0.008 (0.015)	0.524	No Mediation
	HERS-C Resiliency	-0.645 (0.091)***	0.059 (0.108)	0.153 (0.233)	-0.038 (0.070)	-0.541	No Mediation
	HERS-C Safety	0.135 (0.054)*	-0.130 (0.209)	0.153 (0.233)	-0.018 (0.029)	-0.605	No Mediation
	Teacher Efficacy	-2.433 (0.557)***	-0.060 (0.015)***	0.153 (0.233)	0.147 (0.049)**	3.004	Full Mediation
**SDQ Total Problems**							
	Brief Resiliency	-0.460 (0.176)**	-0.155 (0.091)†	1.496 (0.532)**	0.071 (0.050)	1.424	No Mediation
	CWSI Job Control	-0.088 (0.063)	0.297 (0.259)	1.496 (0.532)**	-0.026 (0.030)	-0.883	No Mediation
	CWSI Job Demands	0.156 (0.064)*	0.625 (0.293)*	1.496 (0.532)**	0.098 (0.061)†	1.605	Partial Mediation
	CWSI Job Resources	0.040 (0.043)	-0.895 (0.484)†	1.496 (0.532)**	-0.036 (0.043)	-0.838	No Mediation
	ESI Teacher	0.352 (1.069)	0.031 (0.018)†	1.496 (0.532)**	0.011 (0.034)	0.323	No Mediation
	HERS-C Behavior	-0.168 (0.065)**	-0.706 (0.395)†	1.496 (0.532)**	0.119 (0.081)	1.474	No Mediation
	HERS-C Communication	0.042 (0.072)	0.165 (0.369)	1.496 (0.532)**	0.007 (0.020)	0.354	No Mediation
	HERS-C Resiliency	-0.645 (0.091)***	0.090 (0.237)	1.496 (0.532)**	-0.058 (0.153)	-0.377	No Mediation
	HERS-C Safety	0.135 (0.054)*	-0.813 (0.463)†	1.496 (0.532)**	-0.110 (0.076)	-1.433	No Mediation

Outcome	Mediator	Path A Est. (SE)	Path B Est. (SE)	Direct Effect (Path C) Est. (SE)	Indirect Effect Est. (SE)	Z-Indirect	Mediation Type
	Teacher Efficacy	-2.433 (0.557)***	-0.152 (0.033)***	1.496 (0.532)**	0.371 (0.117)**	3.155	Partial Mediation

Note: Path A = effect of intervention (JS+CS) on mediator; Path B = effect of mediator on outcome; Path C = direct effect of intervention on outcome; DECA = Devereux Early Childhood Assessment (higher scores = better outcomes); SDQ = Strengths and Difficulties Questionnaire (higher scores = more behavior problems); CWSI = Child Care Worker Stress Inventory; HERS-C = Healthy Environments Rating Scale-Classroom; ESI = Everyday Stressors Index—COVID-Related. Standard errors in parentheses.
†$p < .10$. *$p < .05$. **$p < .01$. ***$p < .001$.

Supplementary Table S2. Mediation analysis of JS+CS intervention effects on child outcomes via classroom- and teacher-level factors (change score methods).

Outcome	Mediator	Path A Est. (SE)	Path B Est. (SE)	Indirect Effect Est. (SE)	Z-Score	*p*-value	Total Effect Est. (*p*-value)	Direct Effect Est. (*p*-value)	Mediation Type
**DECA Total Protective Factors**							0.24 (.85)		
	HERS-C Safety	0.36 (0.05)***	-0.11 (1.56)	-0.04 (0.57)	-0.07	.946		1.34 (.39)	No Mediation
	HERS-C Behavior	0.49 (0.05)***	3.55 (1.43)*	1.74 (0.73)*	2.40	.017		-0.38 (.82)	Full Mediation
	HERS-C Communication	0.01 (0.08)	0.63 (1.05)	0.00 (0.05)	0.07	.943		1.26 (.41)	No Mediation
	HERS-C Resiliency	0.75 (0.09)***	-1.55 (0.76)*	-1.16 (0.59)*	-1.97	.049		2.66 (.10)	Full Mediation
	Brief Resiliency	-0.04 (0.22)	-0.16 (0.26)	0.01 (0.04)	0.18	.856		1.03 (.44)	No Mediation
	Teacher Efficacy	0.88 (0.63)	0.29 (0.11)**	0.26 (0.21)	1.24	.215		0.51 (.71)	No Mediation
	CW Job Demands	0.11 (0.07)	-1.05 (1.02)	-0.12 (0.14)	-0.85	.393		0.92 (.49)	No Mediation
	CW Job Resources	-0.10 (0.05)*	-0.05 (1.19)	0.01 (0.12)	0.04	.964		2.08 (.12)	No Mediation
	CW Job Control	0.03 (0.07)	0.65 (0.77)	0.02 (0.05)	0.35	.730		0.98 (.46)	No Mediation

Outcome	Mediator	Path A Est. (SE)	Path B Est. (SE)	Indirect Effect Est. (SE)	Z-Score	*p*-value	Total Effect Est. (*p*-value)	Direct Effect Est. (*p*-value)	Mediation Type
	ESI Teacher	-0.09 (1.25)	-0.00 (0.05)	0.00 (0.01)	0.04	.965		0.71 (.59)	No Mediation
**DECA Attachment/ Relationship**							-1.82 (.14)		
	HERS-C Safety	0.36 (0.05)***	-0.93 (1.60)	-0.34 (0.59)	-0.58	.563		2.36 (.13)	No Mediation
	HERS-C Behavior	0.49 (0.05)***	2.16 (1.46)	1.06 (0.73)	1.46	.145		1.15 (.48)	No Mediation
	HERS-C Communication	0.01 (0.08)	-1.49 (1.03)	-0.01 (0.12)	-0.07	.943		2.21 (.14)	No Mediation
	HERS-C Resiliency	0.75 (0.09)***	-1.55 (0.79)*	-1.16 (0.61)†	-1.92	.055		3.43 (.04)*	No Mediation
	Brief Resiliency	-0.04 (0.22)	-0.49 (0.25)†	0.02 (0.11)	0.19	.849		-0.75 (.57)	No Mediation
	Teacher Efficacy	0.88 (0.63)	0.27 (0.11)*	0.24 (0.20)	1.21	.225		-1.63 (.25)	No Mediation
	CW Job Demands	0.11 (0.07)	-1.42 (1.00)	-0.16 (0.15)	-1.04	.298		-0.89 (.49)	No Mediation
	CW Job Resources	-0.10 (0.05)*	0.42 (1.06)	-0.04 (0.11)	-0.39	.698		0.58 (.65)	No Mediation
	CW Job Control	0.03 (0.07)	0.53 (0.76)	0.01 (0.04)	0.33	.739		-0.88 (.50)	No Mediation
	ESI Teacher	-0.09 (1.25)	0.02 (0.05)	-0.00 (0.03)	-0.07	.941		-1.14 (.38)	No Mediation
**DECA Self-Regulation**							-0.08 (.95)		
	HERS-C Safety	0.36 (0.05)***	1.08 (1.57)	0.39 (0.58)	0.68	.496		-0.43 (.78)	No Mediation
	HERS-C Behavior	0.49 (0.05)***	3.66 (1.43)*	1.79 (0.73)*	2.46	.014		-2.01 (.22)	Full Mediation
	HERS-C Communication	0.01 (0.08)	1.85 (0.99)†	0.01 (0.15)	0.07	.943		-0.41 (.78)	No Mediation
	HERS-C Resiliency	0.75 (0.09)***	-0.81 (0.79)	-0.60 (0.60)	-1.01	.315		0.50 (.76)	No Mediation
	Brief Resiliency	-0.04 (0.22)	0.22 (0.24)	-0.01 (0.05)	-0.19	.852		0.08 (.95)	No Mediation

Outcome	Mediator	Path A Est. (SE)	Path B Est. (SE)	Indirect Effect Est. (SE)	Z-Score	p-value	Total Effect Est. (p-value)	Direct Effect Est. (p-value)	Mediation Type
	Teacher Efficacy	0.88 (0.63)	0.29 (0.10)**	0.25 (0.20)	1.25	.212		-0.20 (.87)	No Mediation
	CW Job Demands	0.11 (0.07)	-0.54 (0.94)	-0.06 (0.11)	-0.53	.593		0.09 (.94)	No Mediation
	CW Job Resources	-0.10 (0.05)*	0.83 (1.06)	-0.08 (0.12)	-0.73	.464		0.96 (.45)	No Mediation
	CW Job Control	0.03 (0.07)	1.10 (0.71)	0.03 (0.08)	0.37	.713		0.21 (.86)	No Mediation
	ESI Teacher	-0.09 (1.25)	0.02 (0.05)	-0.00 (0.02)	-0.07	.942		0.05 (.97)	No Mediation
**DECA Initiative**							1.89 (.10)		
	HERS-C Safety	0.36 (0.05)***	-0.56 (1.47)	-0.20 (0.54)	-0.38	.705		2.59 (.07)†	No Mediation
	HERS-C Behavior	0.49 (0.05)***	2.78 (1.37)*	1.36 (0.69)*	1.98	.048		1.06 (.49)	Partial Mediation
	HERS-C Communication	0.01 (0.08)	1.38 (0.99)	0.01 (0.11)	0.07	.943		2.25 (.11)	No Mediation
	HERS-C Resiliency	0.75 (0.09)***	-1.73 (0.72)*	-1.29 (0.56)*	-2.29	.022		3.93 (.01)**	Partial Mediation
	Brief Resiliency	-0.04 (0.22)	0.07 (0.24)	-0.00 (0.02)	-0.16	.871		2.99 (.01)*	No Mediation
	Teacher Efficacy	0.88 (0.63)	0.29 (0.10)**	0.26 (0.20)	1.27	.205		2.97 (.02)*	No Mediation
	CW Job Demands	0.11 (0.07)	-0.98 (0.92)	-0.11 (0.12)	-0.87	.382		2.99 (.01)*	No Mediation
	CW Job Resources	-0.10 (0.05)*	-1.01 (1.07)	0.10 (0.12)	0.86	.391		3.58 (.00)**	No Mediation
	CW Job Control	0.03 (0.07)	0.19 (0.69)	0.01 (0.02)	0.22	.823		3.02 (.01)*	No Mediation
	ESI Teacher	-0.09 (1.25)	-0.04 (0.05)	0.00 (0.04)	0.07	.941		2.71 (.02)*	No Mediation
**SDQ Total Problems**							-0.13 (.82)		
	HERS-C Safety	0.36 (0.05)***	0.92 (0.75)	0.34 (0.28)	1.20	.230		-0.20 (.77)	No Mediation
	HERS-C Behavior	0.49 (0.05)***	0.72 (0.67)	0.35 (0.33)	1.07	.287		-0.32 (.67)	No Mediation

Outcome	Mediator	Path A Est. (SE)	Path B Est. (SE)	Indirect Effect Est. (SE)	Z-Score	*p*-value	Total Effect Est. (*p*-value)	Direct Effect Est. (*p*-value)	Mediation Type
	HERS-C Communication	0.01 (0.08)	0.43 (0.47)	0.00 (0.03)	0.07	.943		0.02 (.97)	No Mediation
	HERS-C Resiliency	0.75 (0.09)***	0.17 (0.36)	0.13 (0.27)	0.47	.635		-0.10 (.90)	No Mediation
	Brief Resiliency	-0.04 (0.22)	-0.03 (0.12)	0.00 (0.01)	0.16	.874		0.47 (.43)	No Mediation
	Teacher Efficacy	0.88 (0.63)	-0.11 (0.06)†	-0.09 (0.09)	-1.11	.265		0.46 (.47)	No Mediation
	CW Job Demands	0.11 (0.07)	-0.14 (0.44)	-0.02 (0.05)	-0.31	.760		0.47 (.44)	No Mediation
	CW Job Resources	-0.10 (0.05)*	-0.85 (0.48)†	0.09 (0.07)	1.33	.183		0.01 (.98)	No Mediation
	CW Job Control	0.03 (0.07)	0.88 (0.34)*	0.02 (0.07)	0.37	.708		0.51 (.39)	No Mediation
	ESI Teacher	-0.09 (1.25)	-0.02 (0.02)	0.00 (0.02)	0.07	.941		0.47 (.43)	No Mediation
**SDQ Ext. Problems**							-0.31 (.44)		
	HERS-C Safety	0.36 (0.05)***	0.32 (0.53)	0.12 (0.19)	0.60	.548		-0.11 (.82)	No Mediation
	HERS-C Behavior	0.49 (0.05)***	0.09 (0.47)	0.04 (0.23)	0.19	.853		-0.07 (.89)	No Mediation
	HERS-C Communication	0.01 (0.08)	0.08 (0.33)	0.00 (0.01)	0.07	.945		-0.03 (.95)	No Mediation
	HERS-C Resiliency	0.75 (0.09)***	-0.05 (0.26)	-0.04 (0.19)	-0.21	.835		0.02 (.98)	No Mediation
	Brief Resiliency	-0.04 (0.22)	-0.06 (0.08)	0.00 (0.01)	0.19	.853		0.14 (.74)	No Mediation
	Teacher Efficacy	0.88 (0.63)	-0.06 (0.04)	-0.05 (0.05)	-0.97	.331		0.07 (.87)	No Mediation
	CW Job Demands	0.11 (0.07)	0.14 (0.31)	0.02 (0.04)	0.43	.668		0.12 (.77)	No Mediation
	CW Job Resources	-0.10 (0.05)*	-0.42 (0.33)	0.04 (0.04)	1.07	.284		-0.15 (.72)	No Mediation
	CW Job Control	0.03 (0.07)	0.59 (0.24)*	0.02 (0.04)	0.37	.708		0.15 (.71)	No Mediation

Outcome	Mediator	Path A Est. (SE)	Path B Est. (SE)	Indirect Effect Est. (SE)	Z-Score	*p*-value	Total Effect Est. (*p*-value)	Direct Effect Est. (*p*-value)	Mediation Type
**SDQ Int. Problems**	ESI Teacher	-0.09 (1.25)	0.01 (0.02)	-0.00 (0.02)	-0.07	.941	0.18 (.49)	0.12 (.77)	No Mediation
	HERS-C Safety	0.36 (0.05)***	0.60 (0.37)	0.22 (0.14)	1.57	.117		-0.09 (.79)	No Mediation
	HERS-C Behavior	0.49 (0.05)***	0.63 (0.33)†	0.31 (0.17)†	1.87	.061		-0.25 (.50)	No Mediation
	HERS-C Communi cation	0.01 (0.08)	0.35 (0.23)	0.00 (0.03)	0.07	.943		0.05 (.87)	No Mediation
	HERS-C Resiliency	0.75 (0.09)***	0.23 (0.18)	0.17 (0.14)	1.24	.214		-0.11 (.76)	No Mediation
	Brief Resiliency	-0.04 (0.22)	0.03 (0.06)	-0.00 (0.01)	-0.18	.858		0.34 (.24)	No Mediation
	Teacher Efficacy	0.88 (0.63)	-0.05 (0.03)*	-0.05 (0.04)	-1.13	.256		0.39 (.19)	No Mediation
	CW Job Demands	0.11 (0.07)	-0.28 (0.21)	-0.03 (0.03)	-1.00	.318		0.34 (.22)	No Mediation
	CW Job Resources	-0.10 (0.05)*	-0.43 (0.23)†	0.04 (0.03)	1.37	.172		0.17 (.57)	No Mediation
	CW Job Control	0.03 (0.07)	0.29 (0.16)†	0.01 (0.02)	0.37	.711		0.36 (.21)	No Mediation
	ESI Teacher	-0.09 (1.25)	-0.03 (0.01)*	0.00 (0.04)	0.07	.940		0.34 (.22)	No Mediation

Note: Path A = effect of intervention (JS+CS) on mediator; Path B = effect of mediator on outcome; DECA = Devereux Early Childhood Assessment (higher scores = better outcomes); SDQ = Strengths and Difficulties Questionnaire (higher scores = more behavior problems); CWSI = Child Care Worker Stress Inventory; HERS-C = Healthy Environments Rating Scale-Classroom; ESI = Everyday Stressors Index—COVID-Related. All variables represent change scores from baseline to follow-up. Standard errors in parentheses.
†$p < .10$. *$p < .05$. **$p < .01$. ***$p < .001$.

Supplementary Table S3. Generalized estimating equation (GEE) models examining the moderating role of SDQ risk categories on intervention effects for DECA outcomes.

Outcome	Moderator	Term	Estimate (SE)	*p*-value
**DECA Total Protective Factors**	SDQ Total Problems	Treatment Group (JS+CS vs. HC2)	-4.53 (1.02)	<0.001
		Time (Follow-Up vs. Baseline)	0.36 (0.93)	0.697
		SDQ Total Problems (Borderline vs. Normal)	-11.32 (1.68)	<0.001
		SDQ Total Problems (High vs. Normal)	-18.37 (3.95)	<0.001
		SDQ Total Problems (Very High vs. Normal)	-21.48 (2.23)	<0.001
		Treatment Group × Time	1.11 (1.23)	0.366
		Time × SDQ Total Problems (Borderline)	-1.81 (2.32)	0.434
		Time × SDQ Total Problems (High)	7.07 (4.30)	0.100
		Time × SDQ Total Problems (Very High)	5.27 (2.96)	0.075
**DECA Attachment/ Relationships**	SDQ Total Problems	Treatment Group (JS+CS vs. HC2)	-1.53 (1.06)	0.149
		Time (Follow-Up vs. Baseline)	2.92 (0.86)	<0.001
		SDQ Total Problems (Borderline vs. Normal)	-7.65 (2.30)	<0.001
		SDQ Total Problems (High vs. Normal)	-11.74 (2.97)	<0.001
		SDQ Total Problems (Very High vs. Normal)	-16.08 (1.97)	<0.001
		Treatment Group × Time	-0.70 (1.23)	0.571
		Time × SDQ Total Problems (Borderline)	-1.29 (2.84)	0.651
		Time × SDQ Total Problems (High)	3.43 (3.46)	0.322
		Time × SDQ Total Problems (Very High)	4.36 (2.95)	0.139
**DECA Self-Regulation**	SDQ Total Problems	Treatment Group (JS+CS vs. HC2)	-3.81 (0.92)	<0.001
		Time (Follow-Up vs. Baseline)	-1.06 (0.88)	0.230
		SDQ Total Problems (Borderline vs. Normal)	-11.09 (1.79)	<0.001
		SDQ Total Problems (High vs. Normal)	-19.47 (3.24)	<0.001

Outcome	Moderator	Term	Estimate (SE)	*p*-value
		SDQ Total Problems (Very High vs. Normal)	-22.14 (2.16)	<0.001
		Treatment Group × Time	1.31 (1.12)	0.242
		Time × SDQ Total Problems (Borderline)	-2.49 (2.40)	0.300
		Time × SDQ Total Problems (High)	6.28 (3.24)	0.053
		Time × SDQ Total Problems (Very High)	3.41 (2.42)	0.159
**DECA Initiative**	SDQ Total Problems	Treatment Group (JS+CS vs. HC2)	-5.96 (1.02)	<0.001
		Time (Follow-Up vs. Baseline)	-0.86 (1.01)	0.394
		SDQ Total Problems (Borderline vs. Normal)	-9.17 (1.54)	<0.001
		SDQ Total Problems (High vs. Normal)	-17.15 (4.31)	<0.001
		SDQ Total Problems (Very High vs. Normal)	-17.02 (2.12)	<0.001
		Treatment Group × Time	2.59 (1.26)	0.040
		Time × SDQ Total Problems (Borderline)	-1.74 (2.44)	0.476
		Time × SDQ Total Problems (High)	7.80 (4.76)	0.101
		Time × SDQ Total Problems (Very High)	3.51 (2.92)	0.230
**DECA Total Protective Factors**	SDQ Externalizing	Treatment Group (JS+CS vs. HC2)	-2.77 (1.01)	0.006
		Time (Follow-Up vs. Baseline)	-0.99 (0.99)	0.315
		SDQ Externalizing (Borderline vs. Normal)	-4.45 (1.53)	0.004
		SDQ Externalizing (High vs. Normal)	-5.01 (1.44)	<0.001
		SDQ Externalizing (Very High vs. Normal)	-15.83 (1.23)	<0.001
		Treatment Group × Time	0.06 (1.24)	0.964
		Time × SDQ Externalizing (Borderline)	0.03 (2.41)	0.991
		Time × SDQ Externalizing (High)	1.57 (1.79)	0.380
		Time × SDQ Externalizing (Very High)	6.97 (1.63)	<0.001

Outcome	Moderator	Term	Estimate (SE)	*p*-value
**DECA Attachment/ Relationships**	SDQ Externalizing	Treatment Group (JS+CS vs. HC2)	-0.14 (1.05)	0.896
		Time (Follow-Up vs. Baseline)	1.43 (0.97)	0.141
		SDQ Externalizing (Borderline vs. Normal)	-2.23 (1.65)	0.177
		SDQ Externalizing (High vs. Normal)	-5.10 (1.66)	0.002
		SDQ Externalizing (Very High vs. Normal)	-12.10 (1.18)	<0.001
		Treatment Group × Time	-1.20 (1.22)	0.328
		Time × SDQ Externalizing (Borderline)	-2.91 (2.28)	0.201
		Time × SDQ Externalizing (High)	4.03 (2.07)	0.052
		Time × SDQ Externalizing (Very High)	5.76 (1.54)	<0.001
**DECA Self-Regulation**	SDQ Externalizing	Treatment Group (JS+CS vs. HC2)	-1.69 (0.88)	0.054
		Time (Follow-Up vs. Baseline)	-2.26 (0.95)	0.017
		SDQ Externalizing (Borderline vs. Normal)	-3.28 (1.24)	0.008
		SDQ Externalizing (High vs. Normal)	-5.92 (1.10)	<0.001
		SDQ Externalizing (Very High vs. Normal)	-16.68 (1.13)	<0.001
		Treatment Group × Time	0.51 (1.12)	0.650
		Time × SDQ Externalizing (Borderline)	-0.36 (1.88)	0.849
		Time × SDQ Externalizing (High)	1.14 (1.52)	0.454
		Time × SDQ Externalizing (Very High)	5.56 (1.46)	<0.001
**DECA Initiative**	SDQ Externalizing	Treatment Group (JS+CS vs. HC2)	-4.84 (1.01)	<0.001
		Time (Follow-Up vs. Baseline)	-2.31 (1.11)	0.037
		SDQ Externalizing (Borderline vs. Normal)	-4.85 (1.57)	0.002
		SDQ Externalizing (High vs. Normal)	-3.43 (1.42)	0.016
		SDQ Externalizing (Very High vs. Normal)	-12.56 (1.19)	<0.001
		Treatment Group × Time	1.83 (1.28)	0.154

Outcome	Moderator	Term	Estimate (SE)	*p*-value
		Time × SDQ Externalizing (Borderline)	2.72 (2.39)	0.255
		Time × SDQ Externalizing (High)	0.40 (2.06)	0.846
		Time × SDQ Externalizing (Very High)	6.19 (1.55)	<0.001
**DECA Total Protective Factors**	SDQ Internalizing	Treatment Group (JS+CS vs. HC2)	-4.62 (1.02)	<0.001
		Time (Follow-Up vs. Baseline)	-0.12 (0.98)	0.906
		SDQ Internalizing (Borderline vs. Normal)	-5.44 (1.63)	<0.001
		SDQ Internalizing (High vs. Normal)	-9.80 (1.83)	<0.001
		SDQ Internalizing (Very High vs. Normal)	-17.77 (1.85)	<0.001
		Treatment Group × Time	1.22 (1.26)	0.332
		Time × SDQ Internalizing (Borderline)	1.26 (2.38)	0.596
		Time × SDQ Internalizing (High)	0.92 (2.72)	0.736
		Time × SDQ Internalizing (Very High)	4.46 (2.24)	0.046
**DECA Attachment/ Relationships**	SDQ Internalizing	Treatment Group (JS+CS vs. HC2)	-1.54 (1.02)	0.133
		Time (Follow-Up vs. Baseline)	2.05 (0.91)	0.025
		SDQ Internalizing (Borderline vs. Normal)	-7.58 (1.86)	<0.001
		SDQ Internalizing (High vs. Normal)	-6.97 (1.91)	<0.001
		SDQ Internalizing (Very High vs. Normal)	-14.55 (1.57)	<0.001
		Treatment Group × Time	-0.58 (1.21)	0.632
		Time × SDQ Internalizing (Borderline)	3.53 (2.22)	0.112
		Time × SDQ Internalizing (High)	0.79 (2.79)	0.776
		Time × SDQ Internalizing (Very High)	4.97 (2.05)	0.015
**DECA Self-Regulation**	SDQ Internalizing	Treatment Group (JS+CS vs. HC2)	-4.04 (0.98)	<0.001
		Time (Follow-Up vs. Baseline)	-1.30 (0.95)	0.173
		SDQ Internalizing (Borderline vs. Normal)	-2.62 (1.38)	0.058

Outcome	Moderator	Term	Estimate (SE)	*p*-value
		SDQ Internalizing (High vs. Normal)	-8.11 (1.71)	<0.001
		SDQ Internalizing (Very High vs. Normal)	-15.33 (1.84)	<0.001
		Treatment Group × Time	1.45 (1.17)	0.214
		Time × SDQ Internalizing (Borderline)	-1.10 (2.10)	0.602
		Time × SDQ Internalizing (High)	1.33 (2.79)	0.635
		Time × SDQ Internalizing (Very High)	2.58 (2.07)	0.212
**DECA Initiative**	SDQ Internalizing	Treatment Group (JS+CS vs. HC2)	-6.17 (1.00)	<0.001
		Time (Follow-Up vs. Baseline)	-1.16 (1.03)	0.262
		SDQ Internalizing (Borderline vs. Normal)	-4.52 (1.67)	0.007
		SDQ Internalizing (High vs. Normal)	-8.52 (1.70)	<0.001
		SDQ Internalizing (Very High vs. Normal)	-15.46 (1.71)	<0.001
		Treatment Group × Time	3.17 (1.27)	0.012
		Time × SDQ Internalizing (Borderline)	-2.23 (2.56)	0.384
		Time × SDQ Internalizing (High)	0.48 (2.45)	0.845
		Time × SDQ Internalizing (Very High)	3.55 (2.12)	0.094

Note: DECA = Devereux Early Childhood Assessment; SDQ = Strengths and Difficulties Questionnaire. Higher scores on DECA scales indicate better developmental outcomes. SDQ risk categories: normal (within normal range), borderline (slightly elevated), high (high risk), and very high (very high risk). Significant interaction terms ($p < .05$) indicate differential intervention effects based on child risk status.
